# Frequency Band-Specific Electrical Brain Stimulation Modulates Cognitive Control Processes

**DOI:** 10.1371/journal.pone.0138984

**Published:** 2015-09-25

**Authors:** Joram van Driel, Ilja G. Sligte, Jara Linders, Daniel Elport, Michael X Cohen

**Affiliations:** 1 Department of Cognitive Psychology, Vrije Universiteit, Amsterdam, Netherlands; 2 Department of Psychology, University of Amsterdam, Amsterdam, Netherlands; 3 Amsterdam Brain and Cognition, University of Amsterdam, Amsterdam, Netherlands; 4 Visual Experience Lab, Department of Psychology, University of Birmingham, Birmingham, United Kingdom; 5 Science Faculty and University Medical Center, Radboud University, Nijmegen, Netherlands; University Medical Center Goettingen, GERMANY

## Abstract

A large body of findings has tied midfrontal theta-band (4–8 Hz) oscillatory activity to adaptive control mechanisms during response conflict. Thus far, this evidence has been correlational. To evaluate whether theta oscillations are causally involved in conflict processing, we applied transcranial alternating current stimulation (tACS) in the theta band to a midfrontal scalp region, while human subjects performed a spatial response conflict task. Conflict was introduced by incongruency between the location of the target stimulus and the required response hand. As a control condition, we used alpha-band (8–12 Hz) tACS over the same location. The exact stimulation frequencies were determined empirically for each subject based on a pre-stimulation EEG session. Behavioral results showed general conflict effects of slower response times (RT) and lower accuracy for high conflict trials compared to low conflict trials. Importantly, this conflict effect was reduced specifically during theta tACS, which was driven by slower response times on low conflict trials. These results show how theta tACS can modulate adaptive cognitive control processes, which is in accordance with the view of midfrontal theta oscillations as an active mechanism for cognitive control.

## Introduction

In situations of distracting, irrelevant information, multiple competing response alternatives are available. For example, if you are at a cross-road and you need to go left, an arrow-shaped traffic light that points to the right and that suddenly turns green can induce a strong action impulse that needs to be inhibited. This response conflict scenario is an example of how cues in the environment can trigger the need to control our action impulses in order to obtain a desired behavioral goal. Failing to detect response conflict can have serious consequences (e.g. traffic accidents) and is common to various clinical disorders (e.g. AD/HD and Parkinson’s disease; [1]).

Response conflict has been unequivocally shown to coincide with increases in midfrontal theta-band (4–8 Hz) oscillatory activity [2–6], a signature that is thought to be a reflection of neural mechanisms of conflict detection [7,8]. Conflict-related theta most likely originates in posterior, dorsal parts of the medial frontal cortex [9,10], and this region often shows theta phase coupling with other task-relevant regions such as the lateral prefrontal cortex [6,11,12]. Such increases in interareal theta connectivity may reflect orchestrated control signals to improve behavior [13,14].

Although the findings of conflict-related theta are compelling and neurobiologically plausible [7], the body of evidence has thus far been correlational: conflict trials call for increased control, and also produce stronger midfrontal theta activity, compared to non-conflict trials. However, whether this increased theta activity is causally linked to cognitive control of response conflict remains an open question.

Transcranial alternating current stimulation (tACS) can be a useful tool to reveal causal implications of brain oscillations in cognitive function [15,16]. TACS directly injects a current through cortical tissue via two externally placed electrodes, where the direction of current flow alternates between these electrodes in a sinusoidal fashion [17]. This plausibly results in a resonating modulation of membrane potentials of large groups of neurons, and as such entrains oscillatory network activity [18]. Because of this property, tACS provides an intuitive candidate to study conflict-related midfrontal theta dynamics.

Applying tACS in the theta band has thus far been used in other cognitive domains such as working memory [19–21] and intelligence [22], or has served as a control condition [23]. In this study, we applied midfrontal theta tACS while subjects performed a color-location Simon task [24], known to elicit response conflict. As a control frequency, we applied alpha tACS. In contrast to theta, which specifically shows transient conflict-related increases in the time period between stimulus and response, alpha power has been shown to decrease after conflict, which moreover is a more sustained dynamic later in time, when conflict has already been resolved (e.g. in the inter-trial interval; [25,26]). Furthermore, to take into account individual differences [27,28], we used subject-specific peak frequencies for stimulation, based on a pre-stimulation EEG measurement. Our results demonstrate that theta tACS augmented response times specifically towards low-conflict trials, resulting in a relatively reduced conflict effect.

## Materials and Methods

### Subjects

33 subjects (age 19–31; 10 male; all normal or corrected-to-normal vision) from the University of Amsterdam community participated in exchange for 40 euros or course credits. Subjects read an information document about tACS, and gave written informed consent to participate in this study. After the experiment, subjects filled in an exit questionnaire on how they experienced the stimulation (e.g. whether they perceived phosphenes in their visual field). The study and consent procedure was approved by The Faculty Ethics Review Board of the University of Amsterdam, faculty of Social and Behavioral Sciences. All procedures complied with relevant laws and institutional guidelines. Data of two subjects were removed, because of technical problems with the tACS device during stimulation in one of the two sessions. Thus, the final dataset consisted of data from 31 subjects (9 male).

### Task

**Fig 1:**
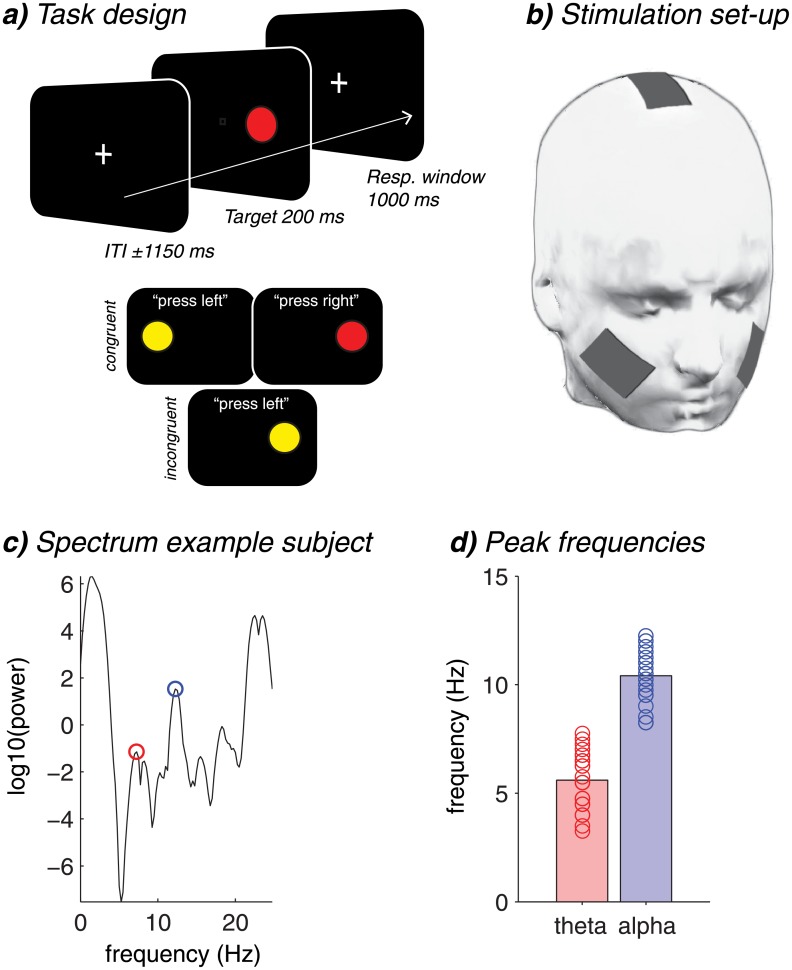
Task design and tACS set-up. a) Trial sequence and timing (top) and example congruent and incongruent trials (bottom) of the Simon task. b) tACS electrodes were placed mid-frontally between Cz and FCz locations (active, 9 cm^2^), and over the two cheeks (reference, each 35 cm^2^). c) EEG power spectrum of one example subject, with the theta (red) and alpha (blue) peak frequencies used for tACS. d) The group average (bars) of used frequencies for theta (red) and alpha (blue) tACS, with the range peak frequencies (open circles).

Subjects performed a color-location Simon task, in which they were instructed to press with, e.g., their left thumb after seeing a blue or yellow circle, and right thumb after seeing a red or green circle. The same colors were used for all subjects and color-response mapping was held constant throughout the task (left vs. right counterbalanced across subjects). Target stimuli (the colored circles) subtended approximately 0.94 degrees in visual angle (dva; given the used eye–screen distance of approximately 100 cm) and appeared for 200 ms approximately 4.69 dva left or right from fixation. Responses were accepted within a window of 1000 ms following target-onset, and trials ended upon responding or when no response was given (in which case feedback with the words “respond faster!” was presented at fixation). The inter-trial interval was on average 1150 ms (jittered across trials between 800 and 1500 ms in steps of 17 ms). A white fixation cross (approximately 0.54 dva) was always present in the center of the screen and all stimuli were presented on a black background. Response conflict was induced by manipulating the correspondence between the location of the target stimulus with the correct response hand. (In)congruent trials were those in which the target appeared in the same (different) visual hemifield as the required response (Fig 1a). In this task, one half of trials was incongruent, and trial type as well as target color and location were pseudo-randomized across trials, such that the same color was never presented more than twice in a row.

### Sessions and blocks

Each subject was invited to the lab on two separate days (across subjects: min. 4, max. 15 days between sessions; M = 7.2). During one of the two sessions, we applied theta tACS, and during the other session we applied alpha tACS, the order of which was counterbalanced across subjects. At the start of both sessions, subjects performed a short practice block of 60 trials. Feedback on accuracy was presented only in this practice block after each trial, with the words “wrong” or “correct.” Feedback was presented at fixation upon responding, for 1000 ms.

Both sessions consisted of three experimental blocks: a pre-stimulation, stimulation, and post-stimulation block. All three blocks consisted of five shorter blocks of 100 trials; between these blocks there were self-paced breaks during which average performance was shown on screen. During the pre-stimulation block of the first session, we measured EEG activity over two channels (FCz and Cz), which corresponded to the location of tACS stimulation (see below for EEG and tACS methodology). During the stimulation block, tACS (theta or alpha) was applied continuously throughout all five 100-trial blocks. After the stimulation block, the tACS equipment was removed and subjects performed a regular post-stimulation block.

### EEG measurement and peak-frequency detection

In order to determine individual peak frequencies within the theta- and alpha-band, we recorded midfrontal EEG activity during the 500 trials of the pre-stimulation block of the first session. For recording, we only used electrodes FCz and Cz of a 64-channel Biosemi ActiveTwo system (biosemi.com) placed according to the international 10–20 system, using both earlobes as reference. Data were sampled online at 2048 Hz. After data acquisition, data were epoched around -1.5 to 2 seconds surrounding stimulus onset, detrended and averaged over the two channels. Only incongruent trials were used for further analyses. To compute the power spectrum during these conflict trials, we applied a fast-Fourier transform (FFT) over 1000 ms sliding windows, ranging from -500 to 1500 around stimulus-onset in 100 ms steps, with 0.25 Hz resolution. To account for edge artifacts the data were tapered prior to FFT using a Hann-taper. Power was log-transformed and detrended (Matlab’s default detrend(x) function, which removes the best straight-line fit) to attenuate the 1/f power scaling, and averaged over the sliding windows and trials. Note that we did not perform rigorous data cleaning such as muscle and eye blink artifact removal. However, these artifacts generally do not contaminate central electrodes such as Cz and FCz.

To determine the subject specific peaks within the theta and alpha band, we used Matlab’s automatic peak detection function findpeaks(x), which returns local maxima in time-varying signals. We applied this function on the power spectra between 3 and 8 Hz to extract a theta peak, and between 8 and14 Hz to extract an alpha peak. The power spectrum from 1 to 25 Hz, with selected values were visually inspected to verify the suggested peak frequencies from the algorithm. This led to some occasional manual changes in detected peaks (3 out of 31 subjects). An example of a power spectrum of one subject, together with the group-average and subject-specific selected peaks, are shown in Fig 1c and 1d. Each subject’s theta and alpha peak are also given in S1 and S2 Files (Supporting Information/Material).

The study reported in this paper is a replication of an earlier study, in which we discovered a bug in the peak-frequency detection algorithm: although every subject was still stimulated in a theta frequency and alpha frequency, this was in most cases not in the subject-specific optimal frequency. Thus, the strength of our intended design (namely, reduce cross-subject variance through subject-specific frequency stimulation) had now introduced a limitation (we unintentionally increased this variance). However, the results of the new dataset highly resembled the results of this previous dataset. We report the main findings of this previous dataset in S1 File (Supporting Information), including two correction analyses that take into account the suboptimal stimulation frequencies.

### TACS settings

We applied tACS (NeuroConn DC-Stimulator MR, neurconn.de) using three rubber electrodes that were attached to the subject’s scalp and skin using Ten20 Conducive Paste (weaverandcompany.com). The target electrode had a size of 9 cm^2^ and was placed over the FCz and Cz location according to the international 10–20 system (the center of the rubber electrode in between FCz/Cz). We used two bigger reference electrodes each of 35 cm^2^ that were placed bilaterally on both cheeks (Fig 1b). We reasoned that this set-up would result in electrical current passing bilaterally through the medial wall of the frontal cortex [29,30]. Stimulation was set to bipolar and sinusoidal, using the detected frequency from the EEG measurement, without DC offset, and ranging between -1 and +1 mA. Stimulation started with a ramp-up of 15 seconds, and was ended manually after task completion, which lasted for ~20 minutes in total. The exact block time depended on average reaction times; for this reason, subjects did not receive a ramp-down (the NeuroConn stimulator requires a fixed duration to be set a priori, after which ramp-down is applied; this was set at 20 minutes but was never reached).

We decided to use alpha stimulation as the control, rather than a sham stimulation condition, because pilot testing revealed that subjects typically know when the stimulation is on (many report feeling a light “buzzing” or “tingling” sensation on the scalp). This contrasts with the typical control condition for tDCS (direct current, non-rhythmic stimulation), for which a ramp-up-and-ramp-down sham is often used. The difference may indicate that subjects are aware of the temporal derivative of the stimulation (which is zero for tDCS and which is always non-zero for tACS). Regardless, it was clear that with our setup, a non-stimulation sham condition would be an unsuitable control condition. In contrast, using alpha as a control frequency ensured that overall physical stimulation was the same across conditions. Moreover, it allowed us to more directly test the frequency-specific hypothesis that theta oscillations play a causal role in conflict processing.

We did not use the beta-frequency range (15–30 Hz) because pilot testing revealed that beta stimulation produced phosphene-like flicker effects in the periphery of the visual field. To evaluate the extent to which this may have also been present during theta and alpha stimulation, we asked subjects after the experiment to rate their experience of phosphenes by drawing a vertical bar on a horizontal line that represented a continuous scale of 0 (no phosphenes) to 10 (strong phosphenes). In a similar way they indicated the extent to which they thought it influenced their performance. Ratings are listed in a supporting table in S1 File (Supporting Information). For theta stimulation, two subjects reported above-zero phosphene experience (1–3.5; influence on performance: 1–3); for alpha, twelve subjects reported above-zero phosphene experience (0.2–5.5; influence on performance: 0–6.5). Although this provides some indication that alpha phosphenes may have introduced an unwanted difference between the two stimulation frequencies, we would like to stress that the majority of subjects did not experience any phosphene-like flicker. Nonetheless, to rule out this potential confound, we recomputed all reported analyses only on those subjects who reported no phosphene experience in either stimulation condition (N = 18); this did not alter the results.

### Statistical analyses

In conflict tasks such as the Simon task, the conflict effect (slower response times or reduced accuracy for incongruent compared to congruent trials) is attenuated when the previous trial was incongruent. This “congruency sequence effect” [31,32] is thought to reflect conflict adaptation. We thus divided trials into whether the current trial was congruent or incongruent (C/I) and whether the previous trial was congruent or incongruent (c/i), resulting in four trial types (cC, iC, cI, iI).

Our main dependent variable of interest concerned reaction time (RT in ms), because this is the most commonly described performance measure in relation to conflict and conflict-sequence effects [33]. To take into account possible speed-accuracy trade-offs, accuracy (% correct) was analyzed as well, together with a behavioral efficiency measure (accuracy divided by RT in seconds) [34]. The results of these complementary analyses are reported in S1 File (Supporting Information). Trials with responses faster than 100 ms were removed. For the average RT per condition, we removed error trials and trials following error trials, because errors happen more often at incongruent trials, are usually faster than correct trials (error speeding), and induce post-error slowing. This may result in biased reaction time differences between conditions related to errors rather than conflict. Post-error slowing and error speeding were analyzed irrespective of conflict condition; these analyses and the main findings are described in S1 File (Supporting Information).

RT was entered into a repeated measures ANOVA with the factors block (pre-stimulation, stimulation, post-stimulation), frequency (theta, alpha), previous congruency (c/i) and current congruency (C/I). As post-hoc analyses, we conducted separate ANOVAs for the theta and alpha session, and additional paired-samples *t*-tests to explore directionality of obtained ANOVA interactions. If the sphericity assumption was violated, we reported the Greenhouse-Geisser correction, with original degrees of freedom.

As a complementary analysis we aimed to quantitatively remove a possible linear practice effect from the RT data, and as such obtain more statistical power in revealing stimulation-specific effects. To this end, we performed for each individual subject a linear least squares fit on the RT data per frequency condition (e.g. the pooled single trials of pre-stimulation, theta stimulation, and post-stimulation), irrespective of conflict condition, with as predictor the block order number (i.e. 1 for pre-stimulation, 2 for stimulation, and 3 for post-stimulation). On the residuals of this linear fitting procedure we performed the same ANOVAs and post-hoc t-tests as described above. The rationale behind this procedure is that the residuals reflect any deviation from a predicted linear decrease in RT due to practicing over time, and are thus more likely to reveal interaction effects between conflict processing and tACS. We also tried a more complete regression model, with three factors: block order, session order (whether theta/alpha stimulation was done in the first/second session), and trial number (assuming linear practice over time within one block); this did not change any of the findings. We report the results of the more simple regression model with only block order as predictor.

All data preprocessing steps (including the least square analysis to obtain residuals, and computation of post-error slowing and error speeding) were performed in Matlab (The Mathworks), and statistical analyses in SPSS (IBM). Matlab scripts as well as SPSS syntax scripts are provided in S2 File (Supporting Material).

## Results

**Fig 2:**
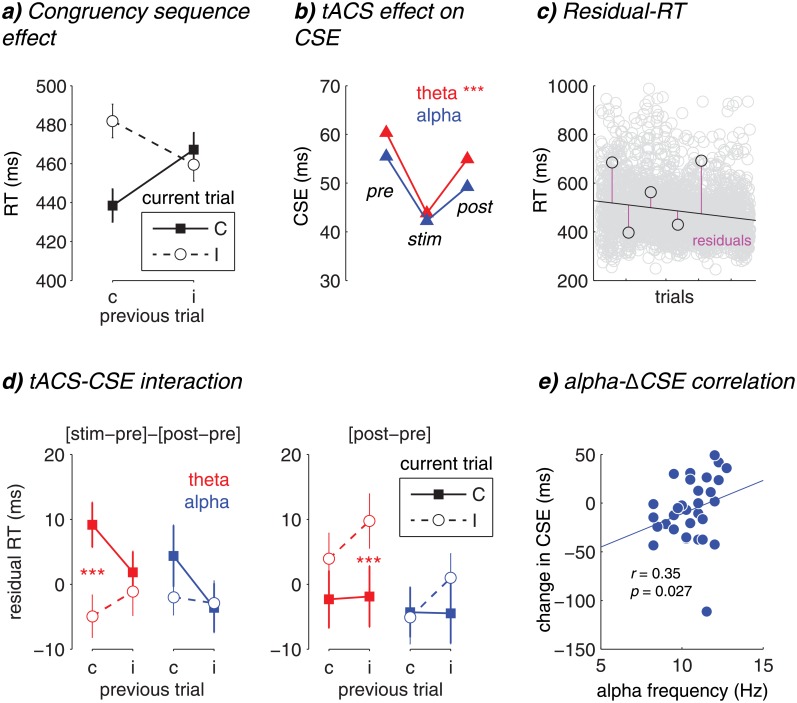
Behavioral results. a) The congruency sequence effect (CSE), showing RT on the current trial (C: congruent, I: incongruent) as a function of previous-trial congruency (c: previous congruent, i: previous incongruent). b) The CSE (defined as [cI–cC]–[iI–iC]) as a function of block (pre, stim, post) and frequency (red: theta, blue: alpha). c) Residual RT calculation. A linear model was fit to all RTs, and the distance from each RT to the linear least square line was taken as the residual RT. d) Left: the effect of tACS (defined as [stim–pre]–[post–pre] on residual RT) plotted as a function of current and previous trial congruency, for theta (red) and alpha (blue) stimulation conditions. Right: similar to left plot, but now the tACS effect was defined as [post–pre]. e) Cross-subject correlation between alpha peak frequency used for stimulation, and the tACS-induced change ([stim–pre]–[post–pre]) in the CSE ([cI–cC]–[iI–iC]). *** p < 0.001.

On average, subjects responded correctly on 91.5% of the trials (SD: 3.51), with a RT of 461.2 ms (SD: 41.91). Collapsed over block and frequency of stimulation, there was a strong conflict effect of increased RT on incongruent compared to congruent trials (*F*_1,30_ = 72.85, *p* < 0.001, *η* ^2^ = 0.71). Moreover, when the previous trial was incongruent this also resulted in slower responses on the current trial (*F*_1,30_ = 8.39, *p* = 0.007, *η* ^2^ = 0.22). Current and previous trial congruency interacted (*F*_1,30_ = 369.1, *p* < 0.001, *η* ^2^ = 0.93), which was explained by a smaller conflict effect after incongruent trials (iI–iC) than after congruent trials (cI–cC; *t*_30_ = 19.21, *p* < 0.001). This effect is known as the “congruency sequence effect” or CSE [32]. As can be seen in Fig 2a, the conflict effect even reversed after incongruent trials (*t*_30_ = 2.84, *p* = 0.008). Thus, the Simon task used in this study elicited the expected trial-by-trial dynamics of conflict detection and adaptation.

To evaluate whether theta-band tACS improved behavior in responding to conflict, we applied the following analysis steps. First, the most robust demonstration of this effect would entail a four-way interaction between stimulation frequency (theta versus alpha), block (pre-stimulation, stimulation, and post-stimulation), current and previous trial congruency. This effect was not obtained (*F*_2,29_ = 0.18, *p* = 0.84, *η* ^2^ = 0.01). However, irrespective of frequency, we found a three-way interaction between block, current and previous trial congruency (*F*_2,29_ = 5.95, *p* = 0.007, *η* ^2^ = 0.29). In other words, the CSE changed as a function of stimulation. We next quantified the CSE by subtracting the conflict effect after incongruent trials from the conflict effect after congruent trials: (cI–cC)—(iI–iC) [33]. Importantly, in this metric positive values reflect a stronger conflict effect on trials following congruent trials compared to trials following incongruent trials). Fig 2b shows the CSE as a function of block and stimulation frequency, where during stimulation a a reduction in CSE can be seen. This effect was significant, as revealed by a quadratic effect of block (*F*_1,30_ = 12.16, *p* = 0.002, *η* ^2^ = 0.29; a linear fit was non-significant: *F*_1,30_ = 2.17, *p* = 0.15, *η* ^2^ = 0.07). Collapsed over frequency, post-hoc dependent samples *t*-tests showed that the CSE was significantly reduced during stimulation compared to pre-stimulation (*t*_30_ = –3.33, *p* = 0.002), and post-stimulation (*t*_30_ = –2.69, *p* = 0.012). There was no difference in CSE between pre- and post-stimulation (*t*_30_ = –1.47, *p* = 0.15). Thus, irrespective of stimulation frequency, we obtained an overall beneficial effect of tACS on behavior during conflict, as reflected by a decreased conflict effect after low-conflict trials (a qualitatively similar pattern of results was obtained for accuracy and behavioral efficiency; details of these analyses are reported in S1 File [Supporting Information]).

We hypothesized a priori that people would benefit in their task performance from theta tACS and not alpha tACS, by showing behavioral improvement in responding to conflict trials specifically only during theta stimulation. Although the four-way interaction was not significant (as mentioned in the previous paragraph), because of our strict hypothesis we conducted a follow-up ANOVA including only the data from the session with theta stimulation. This showed the same three-way interaction between block, previous and current trial congruency (*F*_2,29_ = 7.52, *p* = 0.002, *η* ^2^ = 0.34), with again a quadratic trend of reduced CSE during stimulation (*F*_1,30_ = 15.6, *p* < 0.001, *η* ^2^ = 0.34). In contrast, a similar ANOVA for the alpha session did not show the main interaction (*F*_2,29_ = 2.27, *p* = 0.12, *η* ^2^ = 0.14), although a quadratic trend approached significance (*F*_1,30_ = 3.46, *p* = 0.07, *η* ^2^ = 0.10).

In general, there was a main effect of block on RT (*F*_2,29_ = 26.7, *p* < 0.001, *η* ^2^ = 0.65), which did not interact with frequency (*F*_2,29_ = 1.19, *p* = 0.32, *η* ^2^ = 0.08), and which was best described as a linear trend (*F*_1,30_ = 53.1, *p* < 0.001, *η* ^2^ = 0.64) of decreasing RT over blocks. It is likely that this reflected general training of the task, resulting in faster responding over time. This may have decreased our sensitivity of revealing tACS-conflict interactions “riding on top of” this practice effect. To account for this, we applied a linear least square fitting procedure on the single trial RT data, irrespective of conflict or frequency condition, with block as predictor, and used the residuals as a new dependent variable of interest (Fig 2c). As expected, this removed the main effect (*F*_2,29_ = 1.23, *p* = 0.31, *η* ^2^ = 0.08) and linear trend (*F*_1,30_ = 2.55, *p* = 0.12, *η* ^2^ = 0.08) of block, thus effectively removing practice effects over blocks. Importantly, all interaction effects between CSE and block on these RT-residuals were identical to the above described effects on main RT, confirming stimulation specificity independent from general learning.

Next, on the residuals we computed the stimulation-specific effect through a double subtraction of blocks ([stimulation—pre-stimulation]—[post-stimulation—pre-stimulation]). The reasoning behind this subtraction method is that it effectively removes baseline performance (pre-stimulation) as well as possible after-effects of stimulation (post-stimulation); any conflict-related effect on this metric could then be more confidently attributed to the actual stimulation. Another way of describing this approach is that it captures the quadratic effect of block into one metric, after controlling for the non-specific linear decrease in RT. In Fig 2d (left panel) this tACS-effect is plotted as a function of frequency and previous and current trial congruency, visualizing the interaction between CSE and block on residual RT. A post-hoc t-test revealed that the stimulation-specific effect of theta tACS comprised decreased RT for high conflict trials (cI) compared to increased RT on low conflict trials (cC; *t*_30_ = –3.70, *p* = 0.001). Interestingly, testing this effect for either trial types against zero revealed no significant change from baseline performance on cI trials (*t*_30_ = –1.51, *p* = 0.14), while theta tACS-induced slowing on cC trials was significant (*t*_30_ = 2.66, *p* = 0.01). Importantly, there was no difference between iC and iI trials for theta tACS (|*t*| < 1), and alpha tACS did not modulate any of these conflict-related effects (cI–cC: *t*_30_ = –1.60, *p* = 0.12; iC–iI: |*t*| < 1).

The CSE was defined as a double subtraction ([cI–cC]—[iI–iC]) where the first part (cI–cC) reflects conflict detection (an increased conflict effect after congruent trials), whereas the second part (iI–iC) reflects conflict adaptation (a reduced—or, in this case, reversed—conflict effect after incongruent trials) [32]. The above post-hoc t-tests suggest that theta tACS specifically augmented RT on cC trials, thereby reducing conflict detection costs. Nonetheless, we repeated the above theta- and alpha-specific ANOVAs, now separately for cI versus cC trials (“conflict detection”), and for iI versus iC trials (“conflict adaptation”), again on the residual RTs. This revealed that, as expected, theta tACS modulated conflict detection specifically during stimulation (*F*_2,29_ = 6.97, *p* = 0.003, *η* ^2^ = 0.33; quadratic trend: *F*_1,30_ = 11.46, *p* = 0.002, *η* ^2^ = 0.28), while alpha did not (*F*_2,29_ = 2.07, *p* = 0.15, *η* ^2^ = 0.13). In addition, conflict adaptation changed significantly over blocks for theta (*F*_2,29_ = 9.95, *p* = 0.001, *η* ^2^ = 0.41; linear trend: *F*_1,30_ = 19.01, *p* < 0.001, *η* ^2^ = 0.39), but not alpha (*F*_2,29_ = 1.78, *p* = 0.19, *η* ^2^ = 0.11). Post-hoc t-tests revealed that subjects became slower on iI trials during theta stimulation compared to pre-stimulation (*t*_30_ = 2.33, *p* = 0.027), and remained at this level during post-stimulation (|*t*| < 1). RT on iC trials remained stable over blocks (|*t*| < 1). This effect is visualized in Fig 2d (right panel), where the tACS effect is now defined as [post-stimulation—pre-stimulation]. Note that, because this analysis is done on the RT-residuals after removing the linear decrease in RT, this linear effect on iI trials is due to stimulation, providing evidence for an after-effect of theta tACS irrespective of practice effects over time.

Additionally, our approach allowed us to investigate the individual peak frequencies of stimulation as a potential source of variance in the results. Including individual peak stimulation frequency as a covariate removed the significant CSE-by-block interaction for theta (*F* < 1). In other words, removing variability in stimulation frequency, which we explicitly introduced as an experimental manipulation, also removed the stimulation effect, for theta tACS. However, now the alpha stimulation condition showed a significant quadratic interaction (*F*_2,29_ = 5.35, *p* = 0.028, *η* ^2^ = 0.16). One interpretation of how individual differences in alpha peak frequency may have covaried with the reduction in CSE due to tACS, is that lower alpha frequencies approach the theta band, thus possibly resulting in theta-related behavioral adjustments. To test this hypothesis, we correlated across subjects the alpha tACS modulation of the CSE, with the subject-specific alpha peak frequencies, by computing Spearman’s rho. Given our hypothesis of a positive correlation (the lower the alpha frequency, the stronger the tACS-induced reduction in CSE), we performed a one-tailed test. As expected, we obtained a significant negative correlation (*r*_30_ = 0.35, *p* = 0.027; [*r*_30_ = 0.42, *p* = 0.011 excluding one outlier]; Fig 2e). In other words, removing variability in stimulation frequency, which we explicitly introduced as an experimental manipulation, also removed the stimulation effect.

Taken together, although our results did not show that the conflict-reducing tACS effect was statistically stronger for theta than for alpha, they did show that theta tACS reduced response conflict while alpha tACS did not. However, the theta tACS effect resided in a relatively slower response mode on low conflict (cC) trials during stimulation only, and on conflict trials following earlier conflict (iI) during and after stimulation.

## Discussion

Midfrontal theta dynamics fluctuate together with situations of response conflict [7,8]. This neurocognitive covariance could in theory be epiphenomenal: the theta rhythm may not be part of the actual computations underlying conflict processing, which could be subserved by some other, unknown brain process that may spuriously produce midfrontal theta. Using tACS with a novel stimulation set-up, we obtained evidence supporting an active role of theta oscillations in shaping behavior during response conflict: applying midfrontal tACS with a subject-specific theta-band frequency specifically slowed down response times during low conflict trials, thereby effectively reducing the relative behavioral cost of high conflict trials. This effect was not present when stimulating within the alpha band. To the best of our knowledge, this study is the first to use midfrontal theta tACS in a response conflict task. Two previous studies [29,30], applied tDCS (direct instead of alternating current) over midfrontal sites, and demonstrated that in healthy human subjects, cathodal stimulation (down-regulating cortical excitability) resulted in reduced error- and feedback-related negativity ERP components, as well as improved post-error adaptive behavior, while anodal (up-regulating) stimulation had the exact opposite effect [29]. In schizophrenic patients, this set-up resulted not only in behavioral improvements, but also more synchronized midfrontal theta oscillatory dynamics [30]. In accordance with our findings, this study thus provided similar evidence in favor of a causal role of the medial frontal cortex in performance monitoring, also linking it to frontal theta dynamics [35,36].

### Medial frontal theta-band oscillations causally shape performance during conflict

Although most human electrophysiological evidence of conflict-related theta is correlational in nature, it collectively suggests a causal relationship: conflict-related midfrontal theta power is non-phase locked to stimulus onset and response execution [4], with theta phase synchrony with other task-related regions reflecting functional connectivity [11,37], characterized by strong “hubness” with respect to other scalp regions and frequency bands [38]. Moreover, these manifestations of frontal theta activity predict reaction time at the single-trial level, especially during conflict trials [12]. Our current results provide an important and novel contribution to these findings, by showing more directly that external manipulation of midfrontal theta through tACS alters behavior during conflict trials. Together, these findings are in line with a recently proposed biologically plausible model of a microcircuit of anterior cingulate neurons that generate endogenous theta oscillations [7]. It could be that theta tACS, through rhythmic entrainment, results in a sustained augmentation of this endogenous activity, leading to a more cautious “conflict oriented” slower response mode, which has the biggest effect on *non*-conflict trials. Although this interpretation is post-hoc, a prediction that follows from this is that with concurrently measured EEG during theta tACS, one should observe a reduced conflict-related theta effect (i.e. incongruent relative to congruent trials), primarily driven by relatively stronger midfrontal theta power surrounding the responses on low conflict trials.

The purpose of our pre-stimulation EEG recording was to base the stimulation settings on electrophysiological data. Because we did not measure EEG during or after tACS we cannot definitely prove that altered midfrontal theta oscillations was the intermediate factor driving the behavioral effects. In general, concurrent EEG-tACS studies are important for advancing our understanding about the hypothesized electrophysiological and neurocognitive effects of tACS [16]. Although several studies have provided evidence that tACS can indeed entrain oscillatory activity in the underlying cortical regions [21,30,39,40], this field has far from matured, in comparison to, e.g. neurophysiological effects of TMS [41,42]. Moreover, electrical stimulation studies lack consensus regarding various procedural factors such as appropriate control tasks, control sites, (in the case of tACS) control frequency bands (see Limitations), and stimulation settings such as duration and intensity [41]. These matters require rigorous methodological investigation on their own.

### Conflict detection and adaptation

We replicated the often found trial sequence phenomenon in which conflict-induced slowing at the current trial is greatest when preceded by a non-conflict trial (also known as the “Gratton effect”; [31,32,43]). Moreover, we showed that the conflict effect on RT reversed when preceded by a conflict trial. Such strong conflict adaptation has been interpreted as reflecting active suppression of the irrelevant stimulus dimension following conflict, which hampers action selection during non-conflict trials while facilitating responding to conflict trials [44,45].

During actual stimulation, theta tACS had the strongest effect on conflict detection, mostly through its impact on low-conflict (cC) trials. Interestingly, theta tACS also showed an effect on conflict adaptation: the relative speeding on conflict-following-conflict trials (iI) was reduced during *and* following theta tACS, which might be due to theta tACS resulting in a more cautious response mode. This interpretation can be linked to the view that the medial frontal cortex is sensitive to conflict occurrence, transmitting a conflict signal—which is likely to reside in theta-band oscillatory interactions—to the dorsolateral prefrontal cortex, which then implements increased control for future behavioral improvement [12,13,46,47].

As suggested above, it is possible that constant theta tACS over midfrontal regions results in the medial frontal cortex “evaluating” even low-conflict stimuli as imposing response conflict. Although this is a post-hoc interpretation, it does make several novel predictions that could be empirically tested in follow-up studies. For example, combining our set-up with EEG may show an overall increase in mid-lateral frontal theta phase synchrony. One suggestion for a future study is to apply simultaneous theta tACS both over midfrontal and lateral frontal regions, with the alternating current being either in-phase (inducing increased mid-lateral frontal theta synchrony) or anti-phase (reducing such synchrony), given a common reference electrode (see e.g. [19]). Here, the prediction is that in-phase mid-lateral frontal theta tACS would show the same pattern of results as obtained here (a reduced conflict effect through increased RT on low conflict trials), compared to anti-phase tACS. In addition, given the putative role of the dorsolateral prefrontal cortex in implementing top-down control, in-phase frontal theta stimulation may show increased post-error adaptation effects, such as post-error slowing, which we did not find in our current study (see S1 file [Supporting Information]). In general, this tACS set-up may be well suited to dissociate local midfrontal processing that leads to a conflict signal, from the interareal “broadcasting” of this conflict signal within a frontal network [7].

### Limitations

One recently proposed explanation of conflict-related increases in midfrontal theta-band oscillations is that these simply reflect longer response times (or time-on-task; [48]; see also [49]). This account offers an alternative explanation of our findings, given that augmenting the general level of theta activity in medial frontal cortex would consequently lengthen response times (irrespective of conflict), which could possibly have had a more pronounced effect on the usually faster low-conflict (cC and iI) trials. However, in a reply, [50] argued against this alternative explanation of conflict-related midfrontal theta being a reaction-time epiphenomenon, by showing that many studies that correct for reaction time differences between conflict conditions (e.g., through single-trial regression [12] or trial-selection procedures [36]), still find a robust conflict-related midfrontal theta response. One way of directly testing this account in relation to theta tACS is by including a control condition (e.g. a simple non-conflict target detection task) to test whether theta tACS indeed lengthens response times even in the absence of possible conflict.

Although the conflict-related tACS effect was present only for theta and not for alpha stimulation, we could not make the statistical inference that performance differed between the two stimulation conditions, which would have required a significant four-way interaction. Behavioral effects of tACS are often subtle [51], which may be due to, among other things, limited spatial resolution of the stimulation electrodes, individual differences in cortical folding [52] and cortical excitability [28], or other brain-related parameters that are affected by stimulation but uncontrolled for, such as volume conducted current spread [17,27]. Nonetheless, our results were in accordance with the a priori hypothesis that increased theta activity would reduce response conflict, while alpha would not.

Relatedly, the choice of alpha-band stimulation as a control condition may not have been optimal because of its spectral proximity to the theta band. Furthermore, the selected individual peak frequencies together formed one broad band (see Fig 1d), which may have reduced the sensitivity in finding a significant difference between theta and alpha. Indeed, qualitative inspection of Fig 2 suggests that alpha tACS resulted in similar, though weaker and statistically non-significant effects as theta tACS. This was corroborated by our ANCOVA and follow-up cross-subject correlation analysis, showing that the lower the alpha peak frequency that was used for stimulation, the more stimulating at this frequency reduced the CSE, similar to theta stimulation. In addition, the alpha-band in general may be a suboptimal control frequency, because midfrontal theta and alpha oscillations have been shown to interact through cross-frequency coupling under conditions of response conflict [53]. Using the beta-band (12–30 Hz) as a control condition could provide an alternative to our approach. However, with our current set-up of midfrontal stimulation with cheek electrodes as reference, higher-frequency stimulation resulted in phosphene-like flicker effects in the periphery of the visual field (as evidenced by pilot experiments), which would have introduced unwanted task-irrelevant differences between the stimulation conditions. In general, choosing the appropriate control condition in electrical brain stimulation studies can have various pitfalls and requires careful consideration [16]. Irrespective of the frequency of interest, future studies could explore the possibility to combine tACS with online EEG to better track the effects of tACS on neural oscillations, and to be able to more directly connect these neuroelectrical effects to behavior [39].

## Conclusion

Using theta tACS, we have come closer to answering the yet unresolved question whether midfrontal theta-band oscillatory dynamics are fundamentally involved in response conflict processing. That is, we showed that theta stimulation in particular reduced the relative cost of conflict, by increasing response times on low-conflict situations. In a way, ramping up theta oscillations may result in the medial frontal cortex “detecting” conflict even in the absence of such conflict.

## Supporting Information

S1 File Supporting Information. This document describes three supplementary analyses: 1) a comparison between the RT, accuracy and behavioral efficiency (accuracy divided by RT in seconds); 2) a supplemental analysis on post-error slowing and error speeding; 3) similar analyses as reported in the main text, on an earlier obtained dataset. The document contains a short description of the methods, together with results and accompanying figures.
(PDF)

S2 File Supporting Material; Data and analysis scripts. This zip archive contains the necessary Matlab code, SPSS syntax, SPSS data and raw logfile data to perform the analyses described in the paper. EEG data and Matlab code to extract peak frequencies are available upon request (joramvandriel@gmail.com).
(ZIP)
