# Supplementary material for: Frequency Band-Specific Electrical Brain Stimulation Modulates Cognitive Control Processes
Source: PLoS One. 2015 Sep 25;10(9):e0138984. doi: 10.1371/journal.pone.0138984 (PMC4583279; doi:10.1371/journal.pone.0138984)
Supplement: S1 File — This document describes three supplementary analyses: 1) a comparison between the RT, accuracy and behavioral efficiency (accuracy divided by RT in seconds); 2) a supplemental analysis on post-error slowing and error speeding; 3) similar analyses as reported in the main text, on an earlier obtained dataset. The document contains a short description of the methods, together with results and accompanying figures. (PDF) [file pone.0138984.s001.pdf]

Frequency band-specific electrical brain stimulation modulates cognitive control processes: Supporting information

Joram van Driel[1,2,3,*], Ilja G. Sligte[2,3,4], Jara Linders[2], Daniel Elport[2], and Michael X Cohen[2]

[1] Department of Cognitive Psychology, Vrije Universiteit, Amsterdam Netherlands
[2] Department of Psychology, University of Amsterdam, Amsterdam Netherlands
[3] Amsterdam Brain and Cognition, University of Amsterdam, Amsterdam Netherlands
[4] Visual Experience Lab, Department of Psychology, University of Birmingham, United Kingdom
[*] Corresponding author: joramvandriel@gmail.com

Introduction

This Supporting Information document is part of the paper "Frequency band-specific electrical brain stimulation modulates cognitive control processes". In this paper, data are presented supporting the claim that midfrontal theta-band (4–8 Hz) oscillations have a functional role in conflict processing. In short, we applied theta and alpha (8–12 Hz) transcranial alternating current stimulation over a midfrontal region, with subject-specific peak frequencies, while subjects performed a color-location Simon task. The basic finding was that behavioral costs of response conflict, as quantified by the congruency sequence effect (CSE), was reduced specifically during stimulation, and only for the theta-band condition. In this document we present additional analyses meant to further elucidate and nuance some of our findings.

Reaction time, accuracy, and efficiency

In the main paper we focused our analyses on RT. To complement these findings, we here present the same analyses but now on accuracy and behavioral efficiency [1], in a supplementary Table 1. This latter metric was computed by dividing for each subject and condition, the average accuracy by the average reaction time in seconds. A previous study has shown this metric of task performance to correlate positively with frontal-occipital theta-band connectivity [2]. In terms of cross-subject variance, the efficiency metric ensures that slower but more accurate subjects are equalized to faster but less accurate subjects. However, the same holds for a within-subject difference between conditions: a higher efficiency in condition A than condition B may be driven by 1) more errors in condition B with similar RT in either condition, 2) slower RT in condition B with similar accuracy in either condition, or 3) a mixture of these two.

Based on Table 1, we conclude that theta tACS had its strongest impact on response speed, which is in accordance with results showing a strong (single-trial) relationship with scalp-level midfrontal theta dynamics and reaction time [3–5], and a neurobiologically inspired model of how medial frontal theta oscillations may produce these reaction time fluctuations [6].

tACS effects separately for accuracy, RT, and efficiency

		F	p	η^2	
*block*prev* current*	RT	5.95	0.007	0.29	**
	accuracy	2.79	0.078	0.16	
	efficiency	8.13	0.002	0.36	**
		F	p	η^2	
*block*prev* current THETA*	RT	7.52	0.002	0.34	**
	accuracy	1.08	0.352	0.07	
	efficiency	6.3	0.005	0.31	*
		F	p	η^2	
*block*prev* current ALPHA*	RT	2.27	0.121	0.14	
	accuracy	1.87	0.173	0.11	
	efficiency	2.49	0.100	0.15	
		t	p		
t-test for cC–cI tACS effect THETA	RT	3.70	0.001		***
	accuracy	−1.61	0.119		
	efficiency	−3.68	0.001		***
		t	p		
t-test for cC–cI tACS effect ALPHA	RT	1.60	0.121		
	accuracy	−1.87	0.072		+
	efficiency	−1.96	0.059		+
		t	p		
t-test for iC–iI tACS effect THETA	RT	−4.36	< 0.001		***
	accuracy	2.58	0.015		*
	efficiency	4.06	< 0.001		***
		t	p		
t-test for iC–iI tACS effect ALPHA	RT	−1.55	0.132		
	accuracy	1.09	0.283		
	efficiency	1.54	0.133		

Table 1: Statistical results of repeated measures ANOVAs and post-hoc paired-samples t-tests on three behavioral measures: accuracy, reaction time (RT) and efficiency; the latter is a weighting of accuracy by reaction time in seconds. Reported are the F-values, p-values and effect sizes (η^2) for ANOVAs and t-values and p-values for t-tests. + $p < 0.1$ * $p < 0.05$ ** $p < 0.01$ *** $p < 0.001$

Post-error slowing and error speeding

After committing an error, people usually slow down, a phenomenon known as post-error slowing [7–9]. Post-error slowing is thought to reflect the recruitment of performance adjustment mechanisms, resulting in a more cautious response mode [10]. In contrast, errors are usually characterized by fast responses (error speeding), which is in response-conflict tasks such as the Simon task is thought to be a result of an impulsive response, driven by strong bottom-up processing of the irrelevant stimulus dimension [9, 11, 12]. Given that the reported effects of tACS may reflect a cautious response mode (as expressed in increased RT specifically on cC and iI trials), and given the tight relationship between midfrontal

theta dynamics and error processing [9, 13] it could be that theta tACS, as opposed to alpha tACS also interacted with post-error slowing and error speeding. To test this, we extracted from the behavioral data, for each stimulation condition and irrespective of conflict condition, the residual response times (after removing through a linear least square fitting procedure, the linear effect of decreasing RT over blocks; see main text) of correct trials that were surrounded by two adjacent correct trials (cCc), correct trials that were preceded by error trials (eC), and error trials after correct trials (cE) (see [9]). Residual RTs on these error-related trial types, as a function of block and frequency, are shown in Fig. 1.

First, irrespective of stimulation frequency and block, there was strong post-error slowing, as indicated by slower response times on eC trials than cCc trials ($F[1,30] = 37.88, p < 0.001, \eta^2 = 0.56$). In addition, post-error slowing showed a linear decrease over blocks (accuracy by block interaction: $F[2,29] = 10.69, p < 0.001, \eta^2 = 0.42$; linear trend: $F[1,30] = 15.34, p < 0.001, \eta^2 = 0.34$), which did not further interact with frequency ($F < 0.1$). However, post-hoc t-tests revealed that eC trials decreased in RT over blocks for alpha (pre > post, $t[30] = 3.81, p = 0.001$), while this effect approached significance for theta (t[30] = 1.97, p = 0.058).

Second, the data also exhibited strong error speeding, as indicated by faster response times on cE trials than cCc trials ($F[1,30] = 118.0, p < 0.001, \eta^2 = 0.80$). Error speeding interacted with block ($F[1,30] = 6.36, p = 0.005, \eta^2 = 0.31$), which was best described by a quadratic trend ($F[1,30] = 12.64, p = 0.001, \eta^2 = 0.30$). Moreover, although there was no statistically significant interaction between error speeding, block, and stimulation frequency ($F[1,30] = 2.01, p = 0.15, \eta^2 = 0.12$), here a quadratic trend approached significance ($F[1,30] = 4.14, p = 0.051, \eta^2 = 0.12$). Although this effect should be interpreted with caution, post-hoc t-tests revealed significantly slower response times on cE trials during theta stimulation compared to alpha stimulation ($t[30] = 3.11, p = 0.004$). This result is worth mentioning, because it can also be explained by more cautious (i.e. less impulsive) responding due to midfrontal theta tACS, even when these responses were incorrect.

Individual peak frequencies

In Table 2, each subject's peak frequency for theta and alpha stimulation is shown. EEG data, analysis scripts, and/or spectral power plots of each individual subject are available upon request (joramvandriel@gmail.com).

Complementary results of previous dataset

The study reported in the main text was a replication of an earlier study, in which we discovered a bug in the peak-frequency detection algorithm (see section "EEG measurement and peak-frequency detection"; Materials and Methods): although every subject was still stimulated in a theta frequency and alpha frequency, this was in most cases not in the subject-specific optimal frequency.

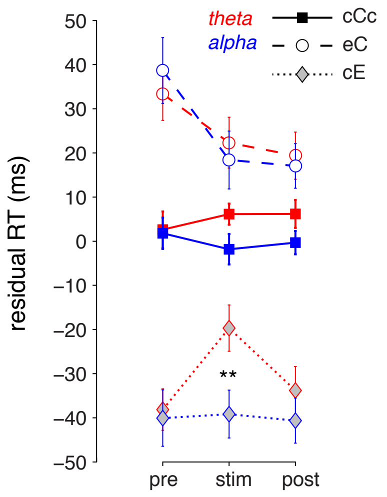

Figure 1: Residual RT (after removing linear trend; see main text) as a function of trial type (cCc, eC, cE), frequency (theta, alpha) and block (pre-stimulation, stimulation, post-stimulation). ** $p < 0.01$.

Thus, the strength of our intended design (namely, reduce cross-subject variance through subject-specific frequency stimulation) had now introduced a limitation (we unintentionally increased this variance). However, the results of the new dataset highly resembled the results of this previous dataset. Because RT and accuracy provided only moderate effects, we focused our analyses of this dataset on the efficiency metric [1].

Materials and Methods

The set-up, task, and basic procedures were identical to the study reported in the main text, except that 30 (different) subjects were tested (age 19–32; 12 male; all normal or corrected-to-normal vision). The study and consent procedure was approved by The Faculty Ethics Review Board of the University of Amsterdam, faculty of Social and Behavioral Sciences. All procedures complied with relevant laws and institutional guidelines. Data from all subjects were included in the final analyses. Another difference is that in this study we did not administer an exit interview questionnaire.

Results

First, this dataset also showed a strong congruency sequence effect (CSE) ($F[1,29] = 184.01, p < 0.001, \eta^2 = 0.86$) of a smaller conflict effect after incongruent trials (iI–iC) than after congruent trials (cI–cC; $t[29] = 13.57, p < 0.001$), where the conflict effect similarly reversed after incongruent trials ($t[29] = 5.75, p < 0.001$). Second, although the four-way interaction between stimulation frequency, block, current and previous trial congruency was absent ($F[2,28] = 0.47, p = 0.63, \eta^2 = 0.03$), the theta-specific ANOVA showed a reduced CSE during stimulation ($F[1,29] = 9.21, p = 0.005, \eta^2 = 0.24$), while this effect was absent for alpha

	Theta			Alpha		
		Phosphene			*Phosphene*	
Subject no.	*Peak (Hz)*	*Strength (0–10)*	*Influence (0–10)*	*Peak (Hz)*	*Strength (0–10)*	*Influence (0–10)*
1	4	0	0	9	0	0
2	6.25	0	0	12.01	2	0
3	4.5	0	0	8.25	0	0
4	5.5	0	0	10.5	0.2	0
5	3.25	0	0	11.01	0	0
6	4.5	0	0	10.26	2.5	2.5
7	6.5	0	0	11.26	2.5	1
8	6.75	0	0	9.5	3	2
9	6.5	0	0	12.01	0	0
10	7.25	1	1	12.26	1	1
11	3.25	0	0	12.76	3	6.5
12	4.75	0	0	11.51	0	0
13	7	0	0	10.51	0	0
14	6.5	3.5	3	12.26	0	0
15	6.75	0	0	10.26	0	0
16	4.75	0	0	11.76	3.5	2
17	5.75	0	0	8.5	0	0
18	3.5	0	0	8.25	0	0
19	3.5	0	0	10.51	0	0
20	4	0	0	11.51	2	3
21	6.75	0	0	11.01	0	0
22	4.75	0	0	10.26	0	0
23	4.5	0	0	9.5	0	0
24	5.5	0	0	11.01	0	0
25	7.75	0	0	10	0	0
26	6.25	0	0	9.5	1	0
27	5.5	0	0	11.01	0	0
28	4	0	0	8.25	0	0
29	6.25	0	0	9.75	0	0
30	7.5	0	0	12.01	5.5	2
31	5.5	0	0	11.26	3	0.5

Table 2: Per frequency condition: Individual peak frequencies used for transcranial alternating current stimulation settings; phosphene ratings.

tACS ($F[1,29] = 1.94, p = 0.18, \eta^2 = 0.06$). This is in strong accordance with our current findings, albeit on a different behavioral metric (Fig. 2ab).

Moreover, post-hoc t-test revealed that the stimulation-specific effect of theta tACS comprised increased behavioral efficiency for high conflict trials (cI) compared to reduced efficiency on low conflict trials (cC; $t[29] = 2.62, p = 0.014$; Fig. 2c), providing similar evidence for a smaller behavioral cost of conflict detection due to theta tACS. However, testing this effect for either trial types against zero could not reveal whether it was better performance on cI trials ($t[29] = 1.04, p = 0.31$), or worse performance on cC trials ($t[29] = -1.56, p = 0.13$) that drove the relative improvement. In this dataset, a main effect of block was weak (theta: $F[2,28] = 2.03, p = 0.15, \eta^2 = 0.07$; alpha: $F[2,28] = 3.0, p = 0.07, \eta^2 = 0.18$), which is evidence against a general learning confound or better general performance during stimulation as a placebo effect.

Conflict adaptation (iI–iC) did show a linear decrease over blocks, here both around theta ($F[1,29] = 8.67, p = 0.006, \eta^2 = 0.23$) and alpha ($F[1,29] = 7.89, p = 0.009, \eta^2 = 0.21$) stimulation. Post-hoc t-tests comparing post-stimulation with pre-stimulation, averaged over the two frequency conditions, revealed that efficiency on iC trials improved ($t[29] = 3.67, p = 0.001$) whereas efficiency on iI trials remained stable over blocks ($t[29] = \breve{\ } 0.30, p = 0.76$). This is somewhat in contrast to our current finding where conflict adaptation was only reduced for theta tACS, driven by slower responses in iI trials.

As noted, the above findings were based on a suboptimal stimulation procedure, because we erroneously used stimulation frequencies that deviated from the subject-specific peak frequencies (Fig. 3a). This may have spuriously introduced, instead of reduced, cross-subject variance in dominant, endogenous peak frequencies [14]. However, with the EEG data we could reliably determine in a post-hoc manner these endogenous peak frequencies (i.e. the frequencies with which we had meant to stimulate our subjects). We could thus introduce the difference between the used (suboptimal) frequency of stimulation and the post-hoc determined (optimal) peak frequency, for theta and alpha separately, as a covariate in the theta- and alpha-specific ANOVAs. These ANCOVAs showed that, after accounting for this difference, the interaction between block, previous and current trial congruency remained significant for theta stimulation, with increased statistical power ($F[1,28] = 16.46, p < 0.001, \eta^2 = 0.37$), while remaining non-significant for alpha ($F[1,28] = 2.02, p = 0.17, \eta^2 = 0.07$). In other words, the reduced congruency sequence effect (CSE) during theta stimulation, which was present even when stimulating in suboptimal frequencies, was statistically more robust after removing this unwanted source of variance. The null-effect of alpha stimulation during conflict, on the other hand, did not seem to depend on the actual frequency used during alpha stimulation.

To further interpret the directionality of these effects,

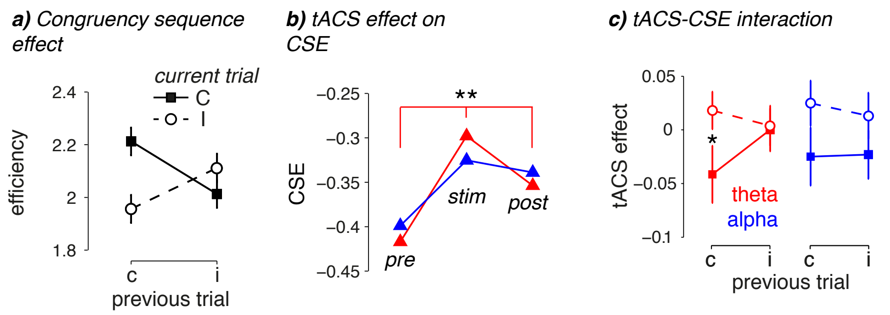

Figure 2: Supplemental behavioral results (earlier dataset). a) The congruency sequence effect (CSE), showing behavioral efficiency (accuracy divided by RT in seconds) on the current trial (C: congruent, I: incongruent) as a function of previous-trial congruency (c: previous congruent, i: previous incongruent). b) The CSE (defined as [cI–cC]–[iI–iC]) as a function of block (pre, stim, post) and frequency (red: theta, blue: alpha). c) The effect of tACS (defined as [stim–pre]–[post–pre]) plotted as a function of current and previous trial congruency, for theta (red) and alpha (blue) stimulation conditions.* $p < 0.05$; ** $p < 0.01$.

we ran a Spearman rank correlation between the absolute difference in applied frequency versus peak frequency, and the CSE during the stimulation block (Fig. 3b). This revealed that, for theta tACS, the closer we stimulated a subject in his/her theta peak frequency, the weaker the CSE (i.e. the stronger the stimulation-induced reduction of the CSE), while subjects that had received a stimulation that was further away from their theta peak showed a stronger CSE (i.e. a weaker stimulation-induced reduction of the CSE) ($r[28] = 0.43, p = 0.018$). This relationship did not hold for the deviance in alpha stimulation from alpha peak frequency, and the CSE during alpha stimulation ($r[28] = 0.23, p = 0.21$).

Conclusion

Together, we believe these two analyses make the general pattern of results of this earlier study more credible. That is, irrespective of the actual frequency of stimulation, subjects did receive theta tACS and alpha tACS nonetheless, which resulted in behavioral improvement towards conflict during theta stimulation. This effect appeared even when using suboptimal frequencies, and increased when accounting for this unwanted variance. Importantly, in the alpha band, we did not find any behavioral improvement, nor did it matter at which exact alpha frequency we stimulated. Moreover, the study reported in the main text provides a powerful replication of the basic pattern of these earlier findings (frequency-specific tACS-induced reduction of conflict costs) in a new group of subjects of similar sample size ($N = 31$), on a better interpretable behavioral metric (RT). The newly acquired dataset furthermore revealed the possible underlying mechanism: midfrontal theta tACS may result in an augmented "conflict detection mode" of cautious responding. Although this explanation is slightly post-hoc, the main text (section Discussion) provides several novel predictions that follow from this interpretation, together with suggestions for future studies that can test these predictions.

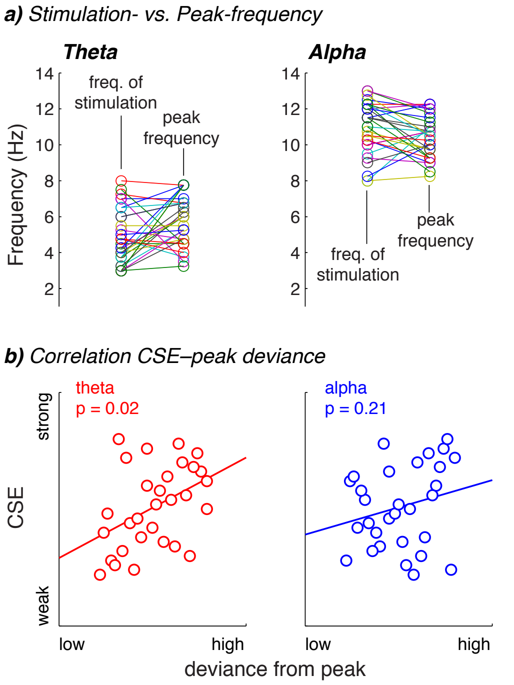

Figure 3: Supplemental correction analysis on incorrect peak-frequency detection in previous dataset. a) Single-subject frequencies used for stimulation versus post-hoc determined peak frequencies, for theta (left panel) and alpha (right panel). Every colored line is one subject. This figure thus shows that for some subjects we stimulated in frequencies close to the endogenous peak frequency, while for other subjects there was a substantial difference between frequencies within a band. b) Spearman rank correlations between the absolute difference between the used frequency and the peak frequency, and the CSE during the stimulation block. For theta stimulation (left panel, red), the closer we stimulated at the peak frequency, the smaller the CSE, pointing towards a subject-specific behavioral benefit of theta tACS during conflict; this relationship did not hold for alpha stimulation (right panel, blue).

Acknowledgements

We thank Joris Oonk and Pia Haver for the data collection of the earlier obtained dataset.

References

1. Townsend, J. T. & Ashby, F. G. *Stochastic Modeling of Elementary Psychological Processes* (Cambridge University Press, UK, 1983).
2. Cohen, M. X., van Gaal, S., Ridderinkhof, K. R. & Lamme, V. A. F. Unconscious errors enhance prefrontal-occipital oscillatory synchrony. *Frontiers in Human Neuroscience* **3,** 54 (2009).
3. Cohen, M. X. & Cavanagh, J. F. Single-trial regression elucidates the role of prefrontal theta oscillations in response conflict. *Frontiers in Psychology* **2,** 30 (2011).
4. Cohen, M. X. & Donner, T. H. Midfrontal conflict-related theta-band power reflects neural oscillations that predict behavior. *Journal of Neurophysiology* **110,** 2752–2763 (2013).
5. Pastötter, B., Dreisbach, G. & Bäuml, K.-H. T. Dynamic Adjustments of Cognitive Control: Oscillatory Correlates of the Conflict Adaptation Effect. *Journal of Cognitive Neuroscience* **25,** 2167–2178 (2013).
6. Cohen, M. X. A neural microcircuit for cognitive conflict detection and signaling. *Trends in Neuroscience* **37,** 480–490 (2014).
7. Ridderinkhof, K. R., Ullsperger, M., Crone, E. A. & Nieuwenhuis, S. The role of the medial frontal cortex in cognitive control. *Science* **306,** 443–447 (2004).
8. Hester, R., Barre, N., Mattingley, J. B., Foxe, J. J. & Garavan, H. Avoiding another mistake: error and posterror neural activity associated with adaptive posterror behavior change. *Cognitive, Affective, & Behavioral Neuroscience* **7,** 317–326 (2007).
9. Van Driel, J., Ridderinkhof, K. R. & Cohen, M. X. Not all errors are alike: theta and alpha EEG dynamics relate to differences in error-processing dynamics. *Journal of Neuroscience* **32,** 16795–16806 (2012).
10. Danielmeier, C. & Ullsperger, M. Post-error adjustments. *Frontiers in Psychology* **2,** 233 (2011).
11. Burle, B & van den Wildenberg, W. Dynamics of facilitation and interference in cue-priming and Simon tasks. *European Journal of Cognitive Psychology* **17,** 619–641 (2005).
12. Ridderinkhof, K. R. Micro- and macro-adjustments of task set: activation and suppression in conflict tasks. *Psychological Research* **66,** 312–323 (2002).
13. Cavanagh, J. F., Cohen, M. X. & Allen, J. J. B. Prelude to and resolution of an error: EEG phase synchrony reveals cognitive control dynamics during action monitoring. *Journal of Neuroscience* **29,** 98–105 (2009).
